# In utero exposure to antiemetic and risk of adult-onset colorectal cancer

**DOI:** 10.1093/jncics/pkad021

**Published:** 2023-03-10

**Authors:** Caitlin C Murphy, Piera M Cirillo, Nickilou Y Krigbaum, Amit G Singal, Barbara A Cohn

**Affiliations:** Department of Health Promotion and Behavioral Sciences, University of Texas Health Science Center at Houston (UTHealth Houston) School of Public Health, Houston, TX, USA; Child Health and Development Studies, Public Health Institute, Berkeley, CA, USA; Child Health and Development Studies, Public Health Institute, Berkeley, CA, USA; Department of Internal Medicine, University of Texas Southwestern Medical Center, Dallas, TX, USA; Harold C. Simmons Comprehensive Cancer Center, Dallas, TX, USA; Child Health and Development Studies, Public Health Institute, Berkeley, CA, USA

## Abstract

**Background:**

Incidence rates of colorectal cancer (CRC) are increasing among adults born in and after the 1960s, implicating pregnancy-related exposures introduced at that time as risk factors. Dicyclomine, an antispasmodic used to treat irritable bowel syndrome, was initially included in Bendectin (comprising doxylamine, pyridoxine, and dicyclomine), an antiemetic prescribed during pregnancy in the 1960s.

**Methods:**

We estimated the association between in utero exposure to Bendectin and risk of CRC in offspring of the Child Health and Development Studies, a multigenerational cohort that enrolled pregnant women in Oakland, CA, between 1959 and 1966 (n = 14 507 mothers and 18 751 liveborn offspring). We reviewed prescribed medications from mothers’ medical records to identify those who received Bendectin during pregnancy. Diagnoses of CRC in adult (aged ≥18 years) offspring were ascertained by linkage with the California Cancer Registry. Cox proportional hazards models were used to estimate adjusted hazard ratios, with follow-up accrued from birth through cancer diagnosis, death, or last contact.

**Results:**

Approximately 5% of offspring (n = 1014) were exposed in utero to Bendectin. Risk of CRC was higher in offspring exposed in utero (adjusted hazard ratio = 3.38, 95% confidence interval [CI] = 1.69 to 6.77) compared with unexposed offspring. Incidence rates of CRC were 30.8 (95% CI = 15.9 to 53.7) and 10.1 (95% CI = 7.9 to 12.8) per 100 000 in offspring exposed to Bendectin and unexposed, respectively.

**Conclusions:**

Higher risk of CRC in offspring exposed in utero may be driven by dicyclomine contained in the 3-part formulation of Bendectin used during the 1960s. Experimental studies are needed to clarify these findings and identify mechanisms of risk.

Incidence rates of colorectal cancer (CRC) are increasing among younger (aged 18-49 years) adults in the United States (1), and more recent evidence suggests rates are also increasing in midlife (aged 50-59 years) (2). Rates of CRC have increased successively by birth cohort (1,3), starting with persons born in the 1960s, therefore renewing interest in identifying risk factors (4-6). Birth cohort effects implicate exposures in early life as risk factors: pregnancy-related exposures introduced in the 1960s may contribute to higher rates of CRC among offspring exposed in utero (7). A well-established experimental literature also supports the importance of gestation for several adult cancers (8-12).

In the 1960s, Bendectin (doxylamine/pyridoxine/dicyclomine) was frequently prescribed to pregnant women to manage nausea and vomiting (13). Bendectin was initially approved in 1956 (14) and quickly became the most common treatment for nausea or vomiting in pregnancy in the United States as its use grew in the 1960s and 1970s (15). After reports of birth defects (16) and concerns in the wake of the thalidomide tragedy (17), in 1976, the manufacturer removed dicyclomine from the 3-part formulation (18). An 8-way randomized trial comparing the relative efficacy of doxylamine, pyridoxine, and dicyclomine suggested no clinical benefit of dicyclomine for nausea or vomiting in pregnancy (19). Production of the 2-part formulation (doxylamine/pyridoxine) was subsequently discontinued in 1983 in the face of ongoing lawsuits (20). Notably, dicyclomine, an antispasmodic (21), continues to be used in clinical practice to treat irritable bowel syndrome and is designated as Pregnancy Category B by the US Food and Drug Administration.

Exposure to Bendectin in utero, and specifically to dicyclomine contained in the 3-part formulation, may directly target the developing gastrointestinal tract of the fetus. This is consistent with some epidemiologic studies demonstrating excess risk of gastrointestinal anomalies (eg, pyloric stenosis, esophageal atresia) in infants of mothers prescribed Bendectin during pregnancy (22-24). Here, we examined the association of in utero exposure to Bendectin and CRC in adult offspring of the Child Health and Development Studies (CHDS), a population-based cohort of more than 18 000 mother-child dyads receiving care in the Kaiser Foundation Health Plan in the 1960s and followed for 60 years. The CHDS has been used extensively to study impacts of early life on health and disease in adulthood and affords the opportunity to link in utero exposures with cancer (25-27).

## Materials and methods

### Study population

The CHDS began in 1959 and recruited nearly all (98%) pregnant women receiving prenatal care from the Kaiser Foundation Health Plan (Oakland, CA, and the surrounding East Bay Area) between June 1959 and September 1966, with deliveries through June 1967. The Kaiser Foundation Health Plan provided care to approximately 30% of the population of Alameda County at that time. A comparison with US census data demonstrated that health plan members—and, by extension, CHDS participants—were demographically similar to the population of the region (28). Additional details of the CHDS are available elsewhere (29,30).

Surveillance of CHDS participants has continued for more than 60 years by linkage to the California Department of Motor Vehicles, California Department of Vital Statistics, and California Cancer Registry. Mothers and their families are matched to these sources using an accumulated name and address history; this cumulative history protects against establishing false matches and failing to identify true matches. At the time of this writing, the majority (64%) of offspring have complete follow-up.

### Primary outcome

We identified diagnoses of CRC in adult (aged ≥18 years) offspring by linkage with the California Cancer Registry through December 31, 2021 (International Classification of Disease in Oncology, 3rd edition codes C18.0-9, C19.9, C20.9). The California Cancer Registry is one of the largest cancer registries in the United States, is gold certified by the North American Association of Central Cancer Registries, and meets the highest-quality data standards set by the National Program of Cancer Registries at the US Centers for Disease Control and Prevention. It is 1 of only 12 state registries funded by both the National Cancer Institute’s Surveillance, Epidemiology, and End Results Program and the US Centers for Disease Control and Prevention’s National Program of Cancer Registries. We used a rigorous protocol to verify cases, comparing fixed (eg, birth date, sex, race) and changeable (eg, address) identifiers by probabilistic matching and manual review. Previous life table analyses in the CHDS have shown close agreement between expected and observed numbers of cases, supporting the accuracy and completeness of cancer ascertainment (31,32).

### In utero exposure to Bendectin

Clinical information, including prenatal visits, diagnosed conditions, and prescribed medications, was prospectively collected from mothers’ medical records beginning 6 months before pregnancy through delivery. All medications are linked to the date and conditions for which they were prescribed. We identified mothers who received Bendectin during pregnancy, including the timing (first trimester: day 0-90; second trimester: day 91-180; third trimester: day ≥181), frequency, and indicating condition.

### Statistical analysis

We used Cox proportional hazards models to estimate the association of in utero exposure to Bendectin and CRC in adult offspring. We used robust estimators to account for nonindependence of observations between siblings (n = 4244). Follow-up time was accrued from date of birth through date of CRC diagnosis, date of death, or date of last contact.

We selected confounders as maternal characteristics associated with both in utero exposure to Bendectin and CRC in adult offspring: year of birth, maternal race (non-Hispanic Black vs else), maternal smoking (current vs else), and maternal body mass index (overweight or obese vs else). Mothers reported demographic and health-related information during in-person interviews at enrollment, including race, smoking, height, and weight. Current smoking was defined as smoking during pregnancy. We used a combination of height and weight reported by mothers during in-person interviews and recorded at the first prenatal visit to measure body mass index (26). As recommended in the literature (33), we did not adjust for mediators or for factors lying on the causal pathway from in utero exposure to CRC, such as birth weight.

We assessed the proportional hazards assumption in adjusted models by including an interaction term of log(time) and in utero exposure. The assumption was not violated (*P* = .16). We report crude and adjusted hazard ratios (aHRs) and 95% confidence intervals (CIs).

We estimated incidence rates and 95% confidence intervals on the basis of the discrete probability distribution for a binomial parameter, separately for offspring exposed and not exposed in utero to Bendectin. Using age as the time scale, we also estimated cumulative incidence of CRC at age 35 years, 40 years, 45 years, 50 years, and 55 years. We estimated cumulative incidence overall and by in utero exposure to Bendectin and compared differences in cumulative incidence by exposure using a log-rank test.

### Sensitivity analyses

To address the possibility of confounding by indication, we examined the association between nausea or vomiting in pregnancy (including hyperemesis gravidarum, nausea gravidarum, morning sickness, nausea of pregnancy, and vomiting of pregnancy) and CRC in adult offspring using Cox proportional hazards models, as detailed above. We also modeled the association of in utero exposure to Bendectin additionally adjusted for nausea or vomiting in pregnancy.

We conducted a probabilistic bias analysis (34,35) to model error from unmeasured confounding. As in our prior studies of the CHDS (25,26), we assigned a trapezoidal distribution for each of 3 bias parameters: 1) prevalence of unmeasured confounder in offspring exposed in utero to Bendectin, 2) prevalence of unmeasured confounder in offspring not exposed, and 3) association between unmeasured confounder and CRC in adult offspring. We repeated the simulation 10 000 times and report the median bias-corrected estimate and 95% simulation interval (additional detail is provided in the Supplementary Material, available online).

Missingness ranged from 1.5% (maternal race) to 20.8% (maternal smoking) for variables included in adjusted models. We used multiple imputation by fully conditional specification to estimate the association between in utero exposure to Bendectin and CRC in adult offspring; fully conditional specification (36) relaxes the assumption of joint multivariable normality and linearity and is well suited for imputation of both categorical and continuous variables.

The institutional review board at the Public Health Institute and the University of Texas Health Science Center at Houston approved this study. Analyses were conducted in SAS version 9.4 (SAS Institute, Cary, NC, USA). All statistical tests were 2-sided, and *P* less than .05 was considered statistically significant.

## Results

Of 18 751 liveborn offspring, 1014 (5.4%) were exposed in utero to Bendectin. Bendectin was most commonly indicated for nausea or vomiting in pregnancy (38.4%) and hyperemesis gravidarum (3.7%). Most offspring were first exposed in the first trimester (73.1%) and to only 1 prescription (86.7%).

Table 1 summarizes characteristics of offspring by in utero exposure. Median follow-up time was similar in offspring exposed (49.5 years; interquartile range (IQR) = 24.5-52.5 years) and not exposed (50.5 years; IQR = 26.5-53.5 years) in utero to Bendectin.

**Table 1.** Characteristics of 18 751 offspring^a^ in the Child Health and Development Studies, 1959-1967, by in utero exposure to Bendectin

	In utero exposure (n = 1014) No. (%)	No in utero exposure (n = 17 737) No. (%)
Offspring characteristics		
Sex		
Male	494 (48.7)	9088 (51.2)
Female	520 (51.3)	8649 (48.8)
Year of birth		
1959-1961	180 (17.8)	5423 (30.6)
1962-1964	486 (47.9)	8559 (48.3)
1965-1967	348 (34.3)	3755 (21.2)
Race and ethnicity		
Asian	39 (3.9)	680 (3.9)
Hispanic	47 (4.7)	566 (3.2)
Mixed race	37 (3.7)	506 (2.9)
Non-Hispanic White	654 (65.0)	11611 (66.5)
Non-Hispanic Black	230 (22.8)	4102 (23.5)
Missing	7	272
Gestational age		
<37 weeks	64 (6.3)	1396 (8.0)
≥37 weeks	950 (93.7)	16043 (92.0)
Missing	0	298
Birth weight, g		
<2500	59 (5.8)	1027 (5.8)
2500-3999	838 (82.6)	15209 (85.8)
≥4000	117 (11.5)	1501 (8.5)
Maternal characteristics		
Maternal age at pregnancy, y		
<20	63 (6.3)	1614 (9.2)
20-24	347 (34.5)	5301 (30.2)
25-29	306 (30.4)	5074 (28.9)
30-34	180 (17.9)	3136 (17.9)
35-39	91 (9.0)	1833 (10.4)
≥40	20 (2.0)	612 (3.5)
Missing	*7*	*167*
Parity at pregnancy		
Primiparous	362 (35.9)	5403 (30.7)
Multiparous	646 (64.1)	12206 (69.3)
Missing	6	128
Body mass index, kg/m2		
Underweight or normal	675 (77.5)	11548 (75.1)
Overweight	156 (17.9)	2849 (18.5)
Obese	40 (4.6)	983 (6.4)
Missing	143	2357
Maternal education		
Less than high school	141 (17.0)	2758 (18.2)
High school or trade school	318 (38.3)	5885 (38.8)
Some college or college degree	372 (44.8)	6521 (43.0)
Missing	183	2573
Maternal smoking^b^		
Never	422 (56.4)	6644 (47.1)
Former	164 (22.0)	2372 (16.8)
Current	161 (21.6)	5088 (36.1)
Missing	267	3633
Annual family income^c^		
≤Median	305 (46.2)	6588 (52.4)
>Median	355 (53.8)	5975 (47.6)
Missing	354	5174

a Live births excluding neonatal deaths to 14 507 mothers.

b Maternal smoking reported during in-person interviews at enrollment; current smoking defined as smoking during pregnancy.

c Median income adjusted to 1960 dollars = $6303.

Over 739 138.5 person-years of follow-up, 83 offspring were diagnosed with CRC in adulthood (Table 2). Approximately 40% (n = 34) of offspring were diagnosed younger than 50 years and the majority with tumors of the distal colon (40.7%) or rectum (32.1%).

**Table 2.** Characteristics of 83 adult offspring diagnosed with CRC

Characteristics	No. (%)
Sex	
Male	40 (48.2)
Female	43 (51.8)
Year of birth	
1959-1961	34 (41.0)
1962-1964	39 (47.0)
1965-1967	10 (12.0)
Race and ethnicity	
Asian	3 (3.8)
Hispanic	5 (6.3)
Mixed Race	4 (5.0)
Non-Hispanic Black	28 (35.0)
Non-Hispanic White	40 (50.0)
Missing	3
Age at diagnosis, y	
Median (IQR)	50 (46 - 53)
18-29	2 (2.4)
30-39	6 (7.2)
40-49	26 (31.3)
50-59	49 (59.0)
Year of diagnosis	
1980-1989	2 (2.4)
1990-1999	4 (4.8)
2000-2009	17 (20.2)
2010-2019	59 (70.2)
2020-2021	2 (2.4)
Stage at diagnosis	
Local	25 (31.3)
Regional	36 (45.0)
Distant	19 (23.8)
Missing	3
Tumor location	
Proximal colon	22 (27.2)
Distal colon	33 (40.7)
Rectum	26 (32.1)
Missing	2
Family history of CRC^a^	
No	71 (85.5)
Yes	12 (14.5)

a Family history of CRC ascertained by linking maternal and paternal records to the California Cancer Registry. CRC = colorectal cancer; IQR = interquartile range.

Offspring exposed in utero to Bendectin had higher risk of CRC (aHR = 3.38, 95% CI = 1.69 to 6.77) compared with offspring not exposed (Table 3). The association was similar in direction and magnitude when using multiple imputation by fully conditional specification (aHR = 3.32, 95% CI = 1.80 to 6.15; Supplementary Table 1, available online) and when using inverse probability of censoring weights to account for the possibility of informative censoring (aHR = 3.25, 95% CI = 1.62 to 6.54; not shown).

**Table 3.** Adjusted hazard ratios and incidence rates (per 100 000 persons) for colorectal cancer in adult offspring with and without in utero exposure to Bendectin

	Person-years	No.	Crude HR (95% CI)	Adjusted HR^a^ (95% CI)	Incidence rate per 100 000 (95% CI)
Bendectin					
Not exposed	700 118.5	71	1.00 (Referent)	1.00 (Referent)	10.1 (7.9 to 12.8)
Any in utero exposure	39 020.0	12	3.66 (1.98 to 6.76)	3.38 (1.69 to 6.77)	30.8 (15.9 to 53.7)

a Adjusted for year of birth, maternal body mass index (overweight or obese vs else), maternal smoking (current vs else), and maternal race (Black vs else).

Observations with missing values (n = 4332) not included in adjusted model (see Supplementary Table 1 available online for results of multiple imputation by fully conditional specification). CI = confidence interval; HR = hazard ratio; Ref = reference category.

Incidence rates of CRC were 30.8 per 100 000 (95% CI = 15.9 to 53.7 per 100 000) and 10.1 per 100 000 (95% CI = 7.9 to 12.8 per 100 000) in offspring exposed and not exposed to Bendectin, respectively (Table 3), corresponding to an incidence rate difference of 20.6 per 100 000 (95% CI = 3.1 to 38.2 per 100 000) and incidence rate ratio of 3.03 (95% CI = 1.58 to 5.45).

**Figure 1.**
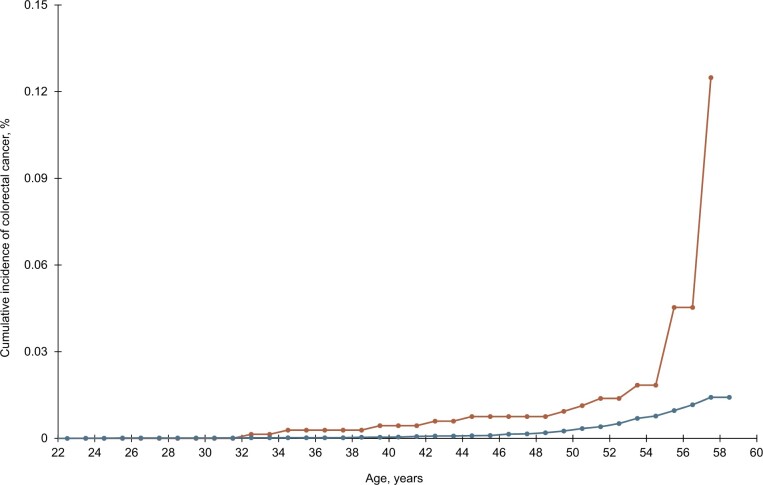
Cumulative incidence of colorectal cancer in adult offspring by in utero exposure to Bendectin. Note: The *x*-axis begins at age 22 years to reflect youngest age at colorectal cancer diagnosis.

As shown in Figure 1, cumulative incidence of CRC also differed by in utero exposure to Bendectin (*P* < .01). For example, at age 50 years, cumulative incidence of CRC was 0.94% (95% CI = 0.42 to 2.08%) and 0.26% (95% CI = 0.18 to 0.37%) in offspring exposed and not exposed, respectively (Supplementary Table 2, available online).

In sensitivity analyses to address unmeasured confounding, there was no association between nausea or vomiting in pregnancy and CRC in offspring (HR = 0.68, 95% CI = 0.31 to 1.48), and the association with in utero exposure to Bendectin remained in a model additionally adjusted for nausea or vomiting in pregnancy (aHR = 4.46, 95% CI = 2.23 to 8.94; not shown). The median bias-corrected estimate from the probabilistic bias analysis (median bias-corrected HR = 2.74, 95% simulation interval = 1.42 to 5.39; Supplementary Material, available online) was similar in direction and magnitude to the observed estimate (aHR = 3.38, 95% CI = 1.69 to 6.77).

## Discussion

In a large multigenerational cohort, we observed an association between in utero exposure to Bendectin and CRC in adult offspring. Incidence rates of CRC were 3 times higher in offspring exposed to Bendectin compared with offspring not exposed. Importantly, offspring birth years (1959-1967) correspond to the years in which Bendectin contained 3 components: doxylamine, an antihistamine; pyridoxine, a form of vitamin B6; and dicyclomine, an antispasmodic. Our findings may reflect a specific effect of dicyclomine or a synergistic effect of the 3 components. As many as 25% of pregnant women received the 3-part Bendectin through the mid-1970s (15), and there may be long-lasting consequences for offspring exposed in utero that continue to present day.

Dicyclomine is both an antispasmodic and anticholinergic agent, and its mechanisms of action may provide clues for understanding the association between Bendectin and CRC in offspring. First, as an antispasmodic, dicyclomine has direct effects on the smooth muscle of the gastrointestinal tract (21). It is possible that in utero exposure programs sensitivity of the developing gastrointestinal tract or increases its susceptibility to carcinogenesis following additional exposures in adulthood. Second, as an anticholinergic agent, dicyclomine inhibits acetylcholine, a neurotransmitter of the parasympathetic nervous system. Acetylcholine receptors are widely distributed in the gastrointestinal tract (37), and dicyclomine may also act synergistically with doxylamine, the antihistamine in Bendectin, because both have anticholinergic effects (38). The developing fetus is exposed to very high concentrations of choline, the precursor to acetylcholine, delivered via the placenta (39,40) to support rapid cell division and growth (41). Maternal choline deficiency during pregnancy results in global and gene-specific DNA methylation (42), and these epigenetic modifications may have lasting effects on metabolic and physiologic processes implicated in cancer (43).

Our findings contribute to the ongoing debate over Bendectin’s teratogenic effects by providing some evidence of carcinogenic effects. Since initial reports of birth defects in the early 1960s (16), Bendectin has been both exonerated from and convicted of teratogenicity. Epidemiologic studies contributing to this debate comprise a range of birth years, making it difficult to disentangle the specific or combined effects of the 2- and 3-part formulations. For example, 2 case-control studies conducted among mothers who likely received the 3-part formulation (birth years 1970-1977) show increased risk of pyloric stenosis (23), esophageal atresia (24), and congenital heart disease (44). Two cohort studies similarly demonstrated elevated although not statistically significantly higher rates of gastrointestinal anomalies in infants of mothers who received Bendectin (45,46). Yet, other studies conducted at this time (47,48), including in the CHDS (49), showed no association with anomalies; however, these studies estimated risk of any anomaly and not specific types. Much of the research later conducted (birth years 1976-1983) was of the 2-part formulation and found no evidence of teratogenicity (50-52). These studies may collectively implicate the 3-part formulation, particularly given its association with gastrointestinal anomalies. The shared etiology of many cancers and birth defects (53) provides additional support.

Prescriptions for Bendectin were prospectively collected from mothers’ medical records and linked by date to the indicating condition, an important strength of our study. Most studies of in utero exposure to Bendectin and birth defects rely on self-report, although 2 used electronic information on prescription fills (22,46). Similarly, CRCs diagnosed in adult offspring were ascertained from a population-based registry, and the robust follow-up of the CHDS affords one of few opportunities to study exposures in utero and cancers diagnosed in adulthood.

Although the timing of the CHDS corresponds to the years in which the 3-part formulation of Bendectin was used, we could not examine the effect of individual components (eg, doxylamine) because very few or no offspring were exposed to only 1 component. Similarly, we could not examine the effect of the timing or frequency of exposure because most exposed offspring were first exposed in first trimester and to only 1 prescription. The association with Bendectin may be confounded by underlying medical conditions, but sensitivity analyses suggest our findings are not due to indications for Bendectin; the median bias corrected estimate from the probabilistic bias analysis was similar, albeit attenuated, to the observed association. Cancers were ascertained in offspring by linkage to the California Cancer Registry, and cancers diagnosed in offspring who have moved away from California are not captured by this linkage. However, the majority of offspring continue to reside in California, and median follow-up time was similar between offspring exposed and not exposed. It is therefore unlikely that differential ascertainment of cancers explains our findings.

In summary, we observed a higher risk of CRC in adult offspring exposed in utero to Bendectin in the 1960s, perhaps driven by dicyclomine contained in the 3-part formulation used during that time. Doxylamine may also potentiate the effect of dicyclomine. Our findings suggest that medications prescribed to pregnant women in the 1960s may, in part, contribute to recent increases in incidence rates of CRC. As the burden of CRC continues to increase in the United States and worldwide (54), well-conducted experimental studies will be critical to clarify these findings and identify mechanisms of risk. Testing for associations with in utero exposure to dicyclomine-containing medications still used during pregnancy may also be warranted.

## Supplementary Material

pkad021_Supplementary_Data

## Data Availability

The data underlying this article cannot be shared in order to protect the privacy and confidentiality of participants who enrolled in the Child Health and Development Studies between 1959 and 1966. Requests for deidentified data will be considered by Barbara A. Cohn, PhD, Director of the Child Health and Development Studies and reviewed by the Institutional Review Board at the Public Health Institute.
