# Supplementary material for: In utero exposure to antiemetic and risk of adult-onset colorectal cancer
Source: JNCI Cancer Spectr. 2023 Mar 10;7(2):pkad021. doi: 10.1093/jncics/pkad021 (PMC10076093; doi:10.1093/jncics/pkad021)
Supplement: pkad021_Supplementary_Data [file pkad021_supplementary_data.pdf]

**Supplementary Table 1. Adjusted hazard ratio for *in utero* exposure to Bendectin and colorectal cancer in adult offspring, using multiple imputation by fully conditional specification for missing values**

| | | | Crude | | Adjusted[1] | |
|---|---|---|---|---|---|---|
| | Person-years | n | HR | 95% CI | HR | 95% CI |
| Bendectin | | | | | | |
| Not exposed | 700,118.5 | 71 | 1.00 | | 1.00 | |
| Any *in utero* exposure | 39,020.0 | 12 | 3.66 | 1.97, 6.77 | 3.32 | 1.80, 6.15 |

Abbreviations: HR, hazard ratio; CI, confidence interval; Ref, reference category

[1]Adjusted for year of birth, maternal body mass index (overweight or obese vs. else), maternal smoking (current vs. else), and maternal race (Black vs. else)

**Supplementary Table 2. Cumulative incidence of colorectal cancer at age 35, 40, 45, 50, and 55 years by *in utero* exposure to Bendectin**

| | | Age 35 y | Age 40 y | Age 45 y | Age 50 y | Age 55 y |
|---|---|---|---|---|---|---|
| | No. | % (95% CI) | % (95% CI) | % (95% CI) | % (95% CI) | % (95% CI) |
| Not exposed | 71 | 0.02 (0.01, 0.07) | 0.04 (0.02, 0.10) | 0.09 (0.05, 0.17) | 0.26 (0.18, 0.37) | 0.78 (0.59. 1.02) |
| Any *in utero* exposure | 12 | 0.29 (0.07, 1.14) | 0.44 (0.14, 1.36) | 0.76 (0.32, 1.81) | 0.94 (0.42, 2.08) | 1.84 (0.90, 3.76) |

**Online Supplement. Probabilistic bias analysis to address unmeasured confounding.**

We conducted a probabilistic bias analysis to model error from unmeasured confounding, assigning a trapezoidal distribution for each of three bias parameters:

- Prevalence of unmeasured confounder in exposed offspring
- Prevalence of unmeasured confounder in unexposed offspring
- Association between unmeasured confounder and colorectal cancer in offspring

We chose a range for the modes of each bias parameter that were reasonable and based on effect estimates reported in the literature (see the World Cancer Research Fund International's 2018 report on colorectal cancer, available at: https://www.wcrf.org/wp-content/uploads/2021/02/Colorectal-cancer-report.pdf); we then extended the trapezoidal distribution to the lower and upper bounds such that the width of the trapezoid was approximately twice the width of the range between modes. We repeated the simulation 10,000 times and report the median bias-corrected HR and 95% simulation interval (corresponding to the $2.5^{th}$ and $97.5^{th}$ percentile of the distribution, including both random and systemic error).

| Bias parameter | Description | Minimum | Mode 1 | Mode 2 | Maximum |
|---|---|---|---|---|---|
| p1 (%) | Prevalence of unmeasured cofounder in offspring exposed *in utero* to Bendectin | 60 | 65 | 75 | 80 |
| p0 (%) | Prevalence of unmeasured confounder in offspring not exposed | 20 | 25 | 35 | 40 |
| $RR_{CD}$ | Association between unmeasured confounder and colorectal cancer in offspring | 0.9 | 1.2 | 1.7 | 2.0 |

As shown in the table below, the median bias corrected-HRs from all simulations were slightly attenuated from but similar to the observed aHRs.

*In utero* exposure to Bendectin (exposed vs. not exposed)

| | Adjusted HR | 95% confidence interval |
|---|---|---|
| Observed | 3.38 | 1.69, 6.77 |

| | Median bias-corrected HR | 95% simulation interval |
|---|---|---|
| Systemic error | 2.72 | 2.38, 3.13 |
| Systemic and random error | 2.74 | 1.42, 5.39 |
